# Supplementary material for: The genetic landscape of basal ganglia and implications for common brain disorders
Source: Nat Commun. 2024 Oct 1;15:8476. doi: 10.1038/s41467-024-52583-0 (PMC11445552; doi:10.1038/s41467-024-52583-0)
Supplement: Supplementary file 1 — Supplementary Information [file 41467_2024_52583_MOESM1_ESM.pdf]

Supplementary Information

Unveiling the Genetic Landscape of Basal Ganglia: Implications for Common Brain Disorders

Shahram Bahrami (Ph.D.)[1,2#*], Kaja Nordengen (M.D. Ph.D.)[1,3#], Jaroslav Rokicki (Ph.D.)[4], Alexey A. Shadrin (Ph.D.)[1,2], Zillur Rahman (Ph.D.)[2], Olav B. Smeland (M.D. Ph.D.)[1], Piotr P. Jaholkowski (M.D. Ph.D.)[1], Nadine Parker (Ph.D.)[1], Pravesh Parekh (Ph.D.)[1], Kevin S. O'Connell (Ph.D.)[1], Torbjørn Elvsåshagen (M.D. Ph.D.)[1,3,5], Mathias Toft (M.D. Ph.D.)[1,3], Srdjan Djurovic (Ph.D.)[1,6], Anders M. Dale (Ph.D.)[7-10], Lars T. Westlye (Ph.D.)[1,11], Tobias Kaufmann (Ph.D.)[1,12,13], Ole A. Andreassen (M.D. Ph.D.)[1,2, 14*]

# equal contribution

1. Institute of Clinical Medicine, University of Oslo, Oslo, Norway
2. KG Jebsen Centre for Neurodevelopmental disorders, University of Oslo, Oslo, Norway
3. Department of Neurology, Oslo University Hospital, Oslo, Norway
4. Centre of Research and Education in Forensic Psychiatry, Oslo University Hospital, Oslo, Norway
5. Department of Behavioral Medicine, Institute of Basic Medical Sciences, University of Oslo, Oslo, Norway
6. Department of Medical Genetics, Oslo University Hospital, Oslo, Norway
7. Multimodal Imaging Laboratory, University of California San Diego, La Jolla, USA
8. Department of Psychiatry, University of California, San Diego, La Jolla, USA,
9. Department of Neurosciences, University of California San Diego, La Jolla, USA
10. Department of Radiology, University of California, San Diego, La Jolla, USA
11. Department of Psychology, Faculty of Social Sciences, University of Oslo, Norway
12. Department of Psychiatry and Psychotherapy, Tübingen Center for Mental Health, University of Tübingen, Germany
13. German Center for Mental Health (DZPG), Germany
14. Department of Psychiatry, Oslo University Hospital, Oslo, Norway

Table of content Supplementary item Page

1 Supplementary Fig. 1 ………………. 3

2 Supplementary Fig. 2 ………………. 4

3 Supplementary Fig. 3 ………………. 5

4 Supplementary Fig. 4 ………………. 6

5 Supplementary Fig. 5 ………………. 7

6 Supplementary Fig. 6 ………………. 8

7 Supplementary Fig. 7 ………………. 9

8 Supplementary Fig. 8 ………………. 10

9 Supplementary Fig. 9 ………………. 11-13

10 Supplementary Fig. 10 ……………. 14

11 Supplementary Fig. 11 ……………. 15

12 Supplementary Fig. 12 ……………. 16

13 Supplementary Fig. 13 ……………. 17

14 Supplementary Fig. 14 ……………. 18

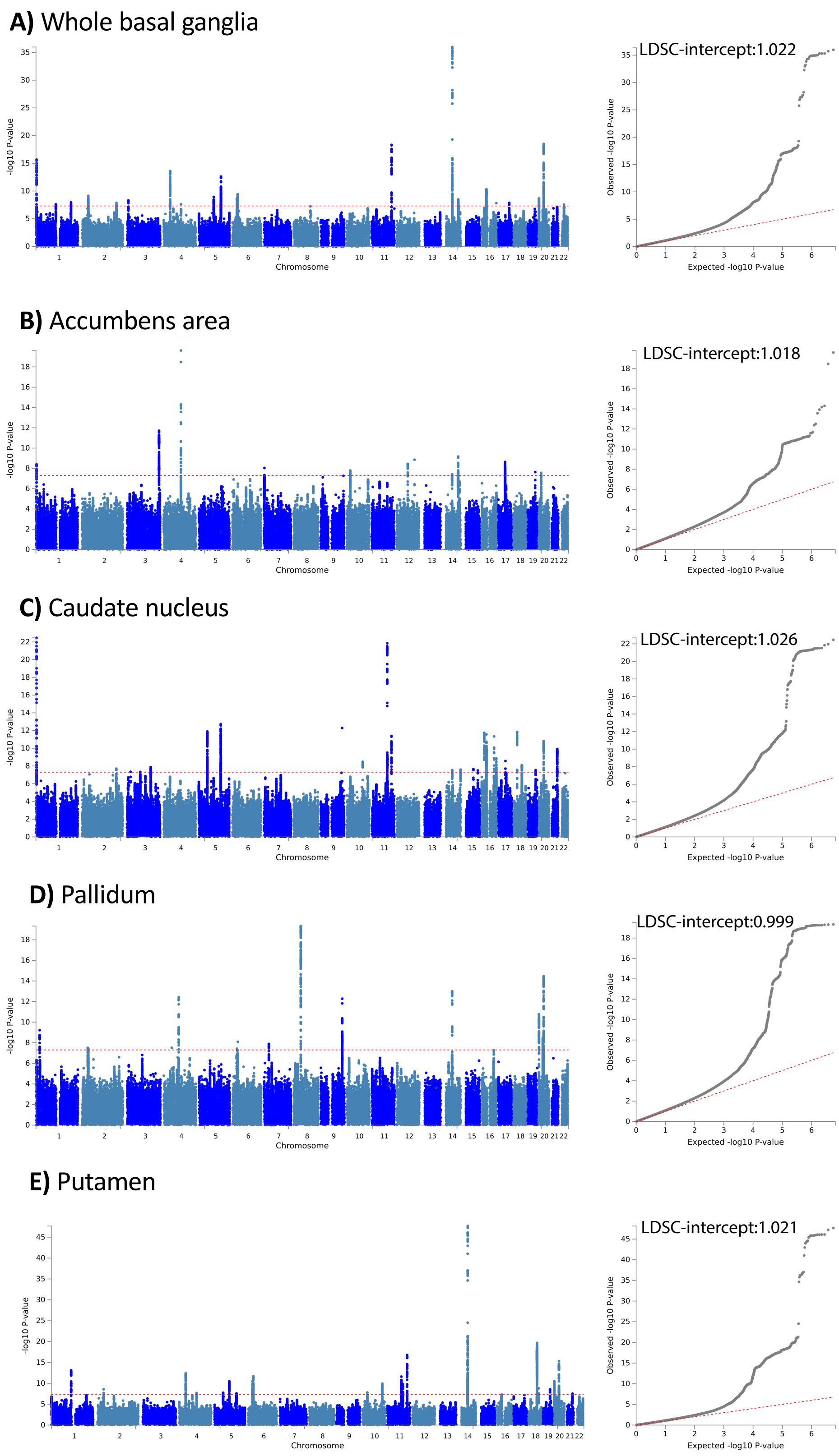

Supplementary Fig. 1. Manhattan and QQ-plots for univariate GWASs on basal ganglia (A), and the individual nuclei: accumbens area (B), caudate nucleus (C), pallidum (D) and putamen (E).. Each plot also provides the intercept from LD-score regression analyses (LDSC-intercept), where values close to 1 indicate no or minimal inflation due to bias (such as cryptic relatedness and population stratification). Manhattan- and QQ-plots from FUMA (https://fuma.ctglab.nl/).

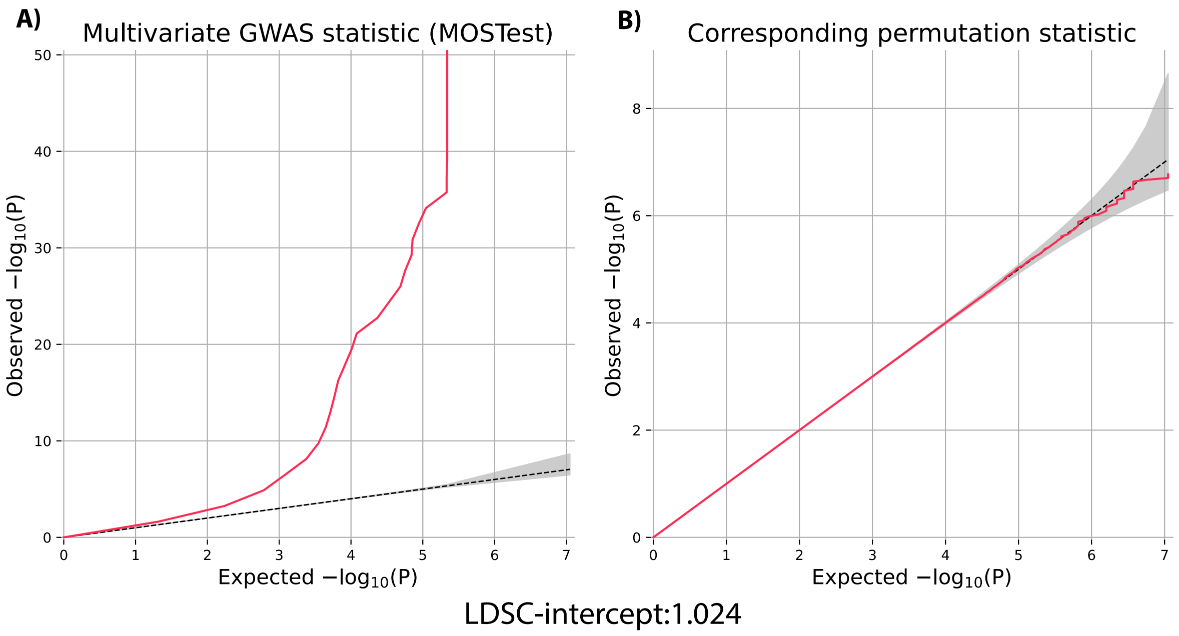

Supplementary Fig. 2. Quantile-quantile (Q-Q) plots from MOSTest analysis. **A)** The left panel shows signal from MOSTest analysis. **B)** The right panel (from permutation testing) shows test statistics under null and confirms validity of the MOSTest test statistics.

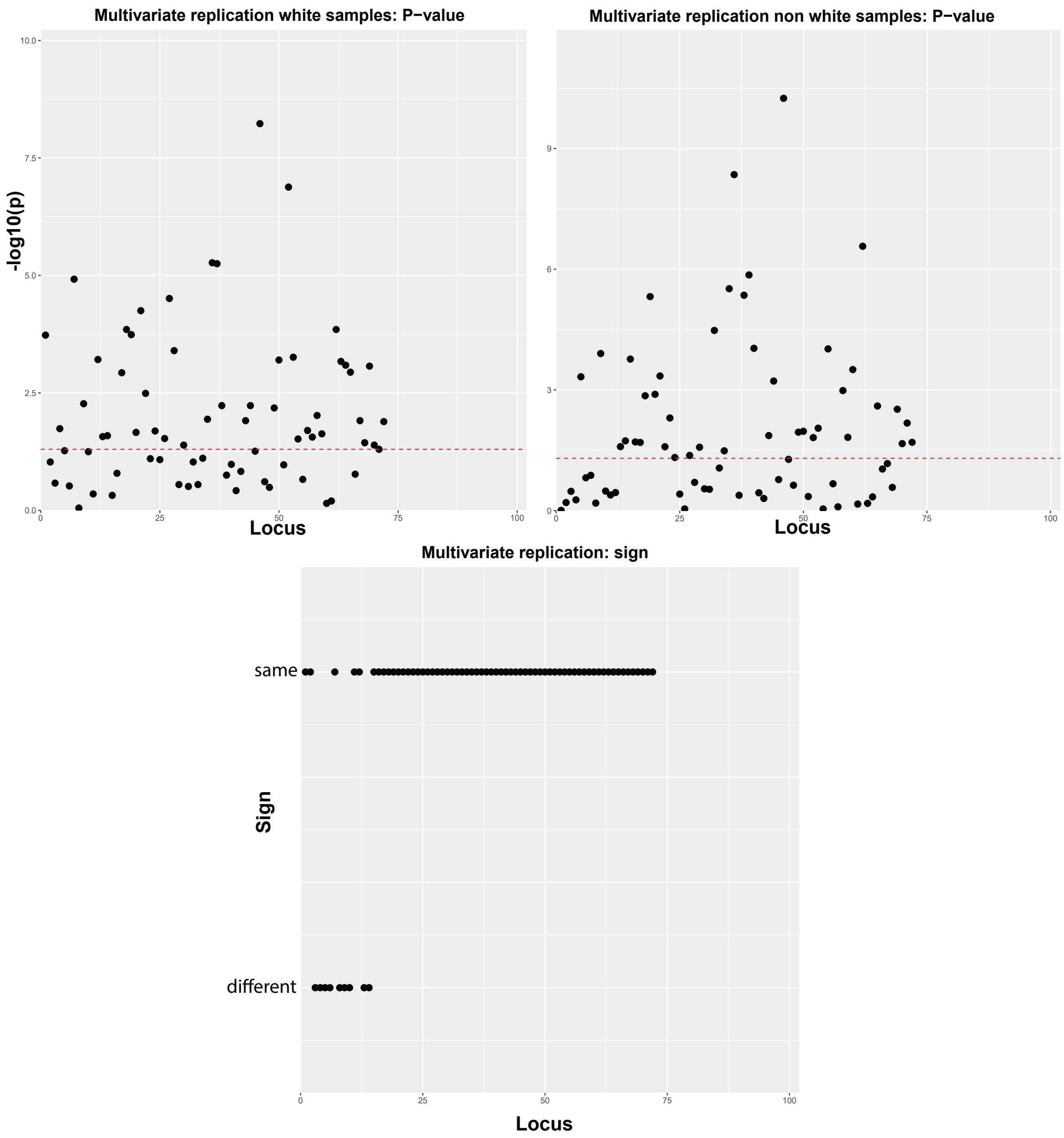

Supplementary Fig. 3. Two multivariate replication analyses using independent data from 4808 white people and 5220 individuals with non-white ethnicity (generalization). Using a multivariate replication procedure (see Methods), we found that 55.6% of the loci replicated at $P < 0.05$ and 87.5% showed the same direction. P-values are denoted as -log10(P).

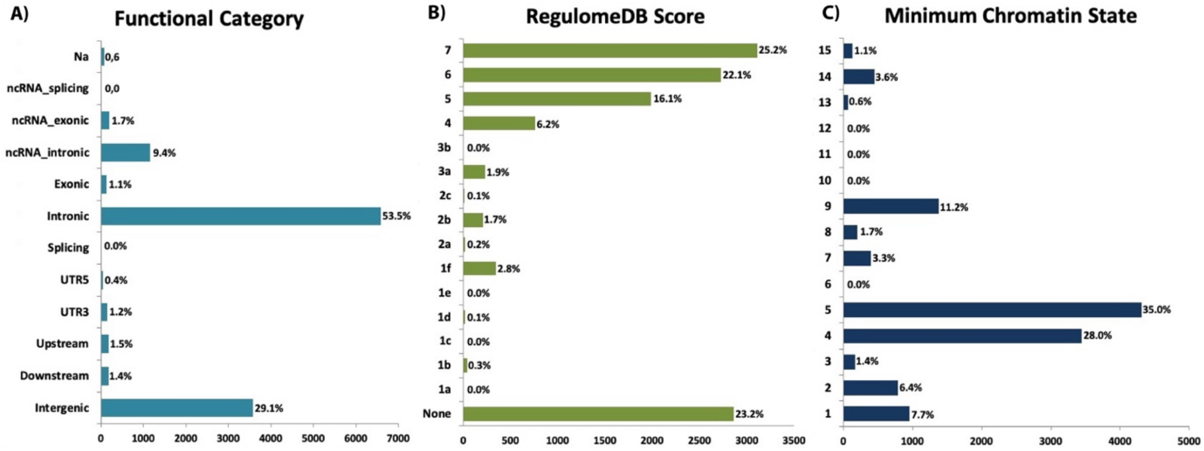

Supplementary Fig. 4. Distribution of the annotation for all SNPs in the significant genetic loci from the basal ganglia GWAS including (A) the minimum chromatin state across 127 tissue and cell types for SNPs in the significant genomic loci, with lower states indicating higher accessibility and states 1–7 referring to open chromatin states, (B) the distribution of RegulomeDB scores for SNPs in the significant genomic loci, with a low score indicating a higher likelihood of having a regulatory function and (C) the distribution of functional consequences of SNPs in the significant genomic risk loci. The chromatin states are 1=Active Transcription Start Site (TSS); 2=Flanking Active TSS; 3=Transcription at gene 5' and 3'; 4=Strong transcription; 5=Weak Transcription; 6=Genic enhancers; 7=Enhancers; 8=Zinc finger genes & repeats; 9=Heterochromatic; 10=Bivalent/Poised TSS; 11=Flanking Bivalent/Poised TSS/Enh; 12=Bivalent Enhancer; 13=Repressed PolyComb; 14=Weak Repressed PolyComb; 15=Quiescent/Low. RegulomeDB categories reflect: 1a: eQTL + TF binding + matched TF motif + matched DNase Footprint + DNase peak; 1b: eQTL + TF binding + any motif + DNase Footprint + DNase peak; 1c: eQTL + TF binding + matched TF motif + DNase peak; 1d: eQTL + TF binding + any motif + DNase peak; 1e: eQTL + TF binding + matched TF motif; 1f: eQTL + TF binding / DNase peak; 2a: TF binding + matched TF motif + matched DNase Footprint + DNase peak; 2b: TF binding + any motif + DNase Footprint + DNase peak; 2c: TF binding + matched TF motif + DNase peak; 3a: TF binding + any motif + DNase peak; 3b: TF binding + matched TF motif; 4: TF binding + DNase peak; 5: TF binding or DNase peak; 6: Motif hit; 7: Other.

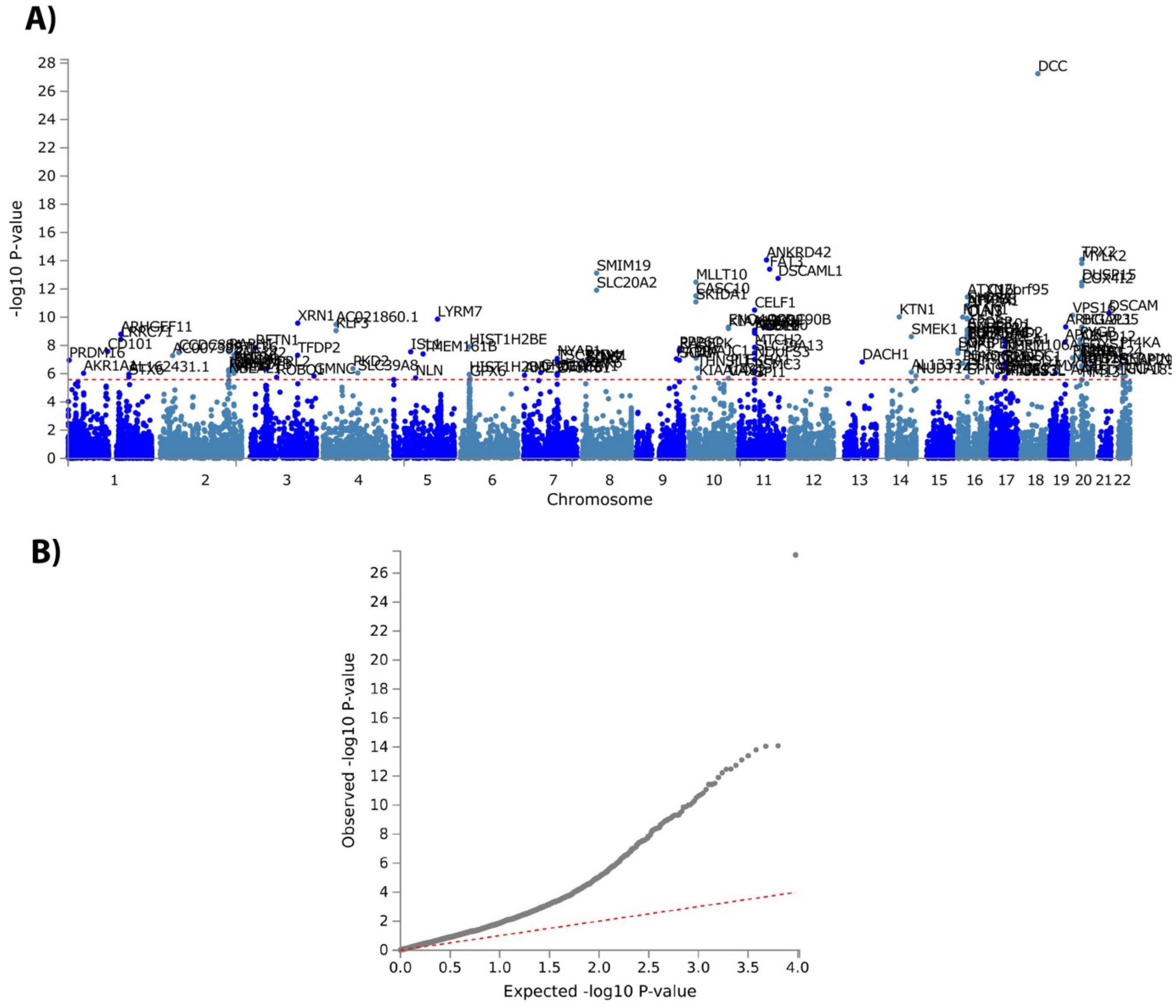

Supplementary Fig. 5. A. Manhattan plots from the genome-wide gene-based association analyses for basal ganglia. 149 genes were associated with basal ganglia. The red horizontal lines indicate significance threshold of two-sided P = 2.622e-6 . **B.** That is a Q-Q plot of the gene-based test computed by MAGMA. Manhattan- and QQ-plots from FUMA (https://fuma.ctglab.nl/).

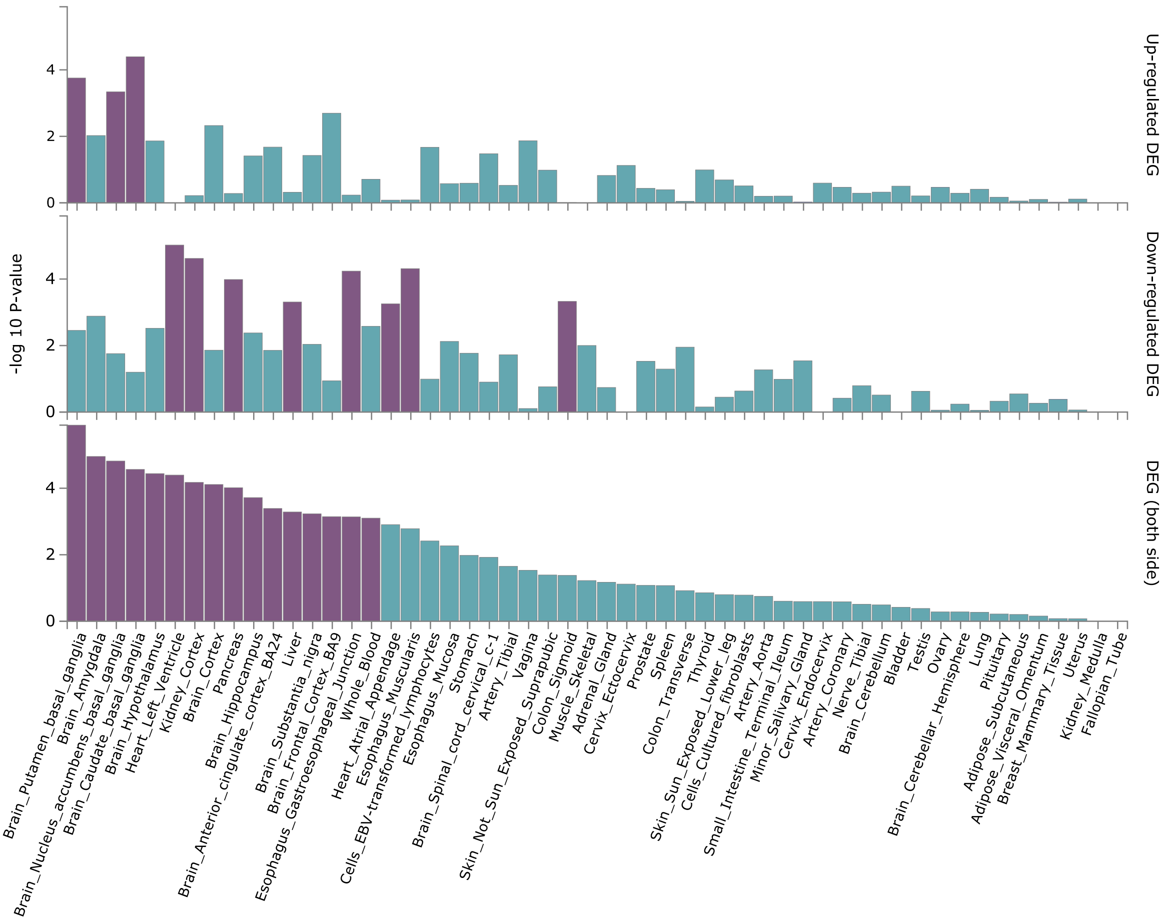

Supplementary Fig. 6. GTEx enrichment analysis based on the 75 genes mapped by Open target. General tissue types.

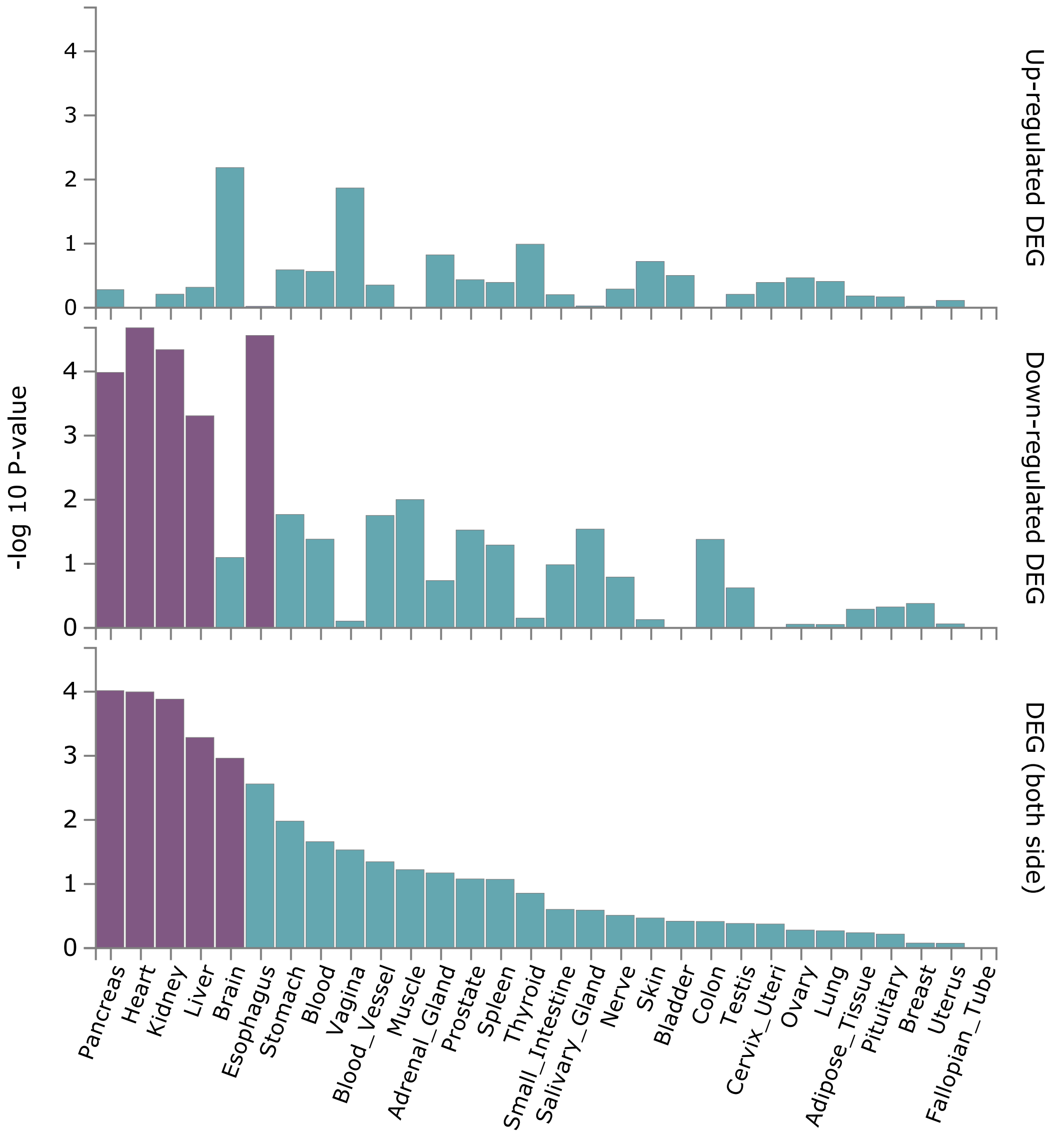

Supplementary Fig.7. GTEx enrichment analysis based on the 75 genes mapped by Open target. Tissue types. P-values are two-tailed.

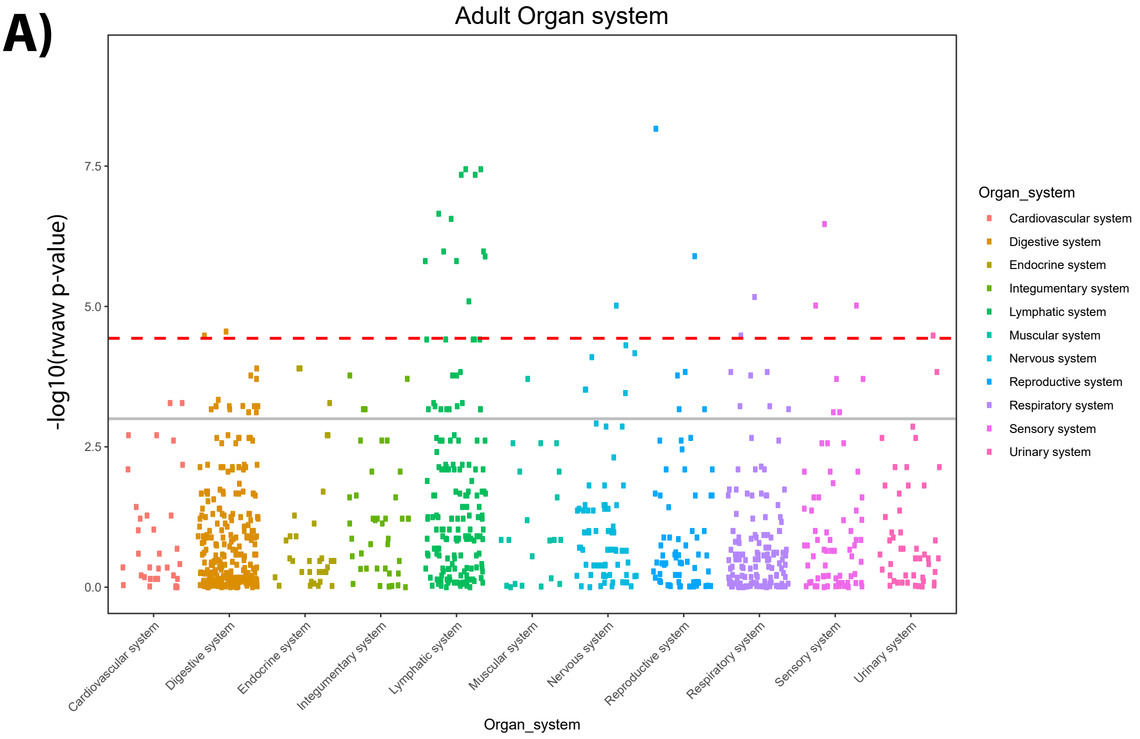

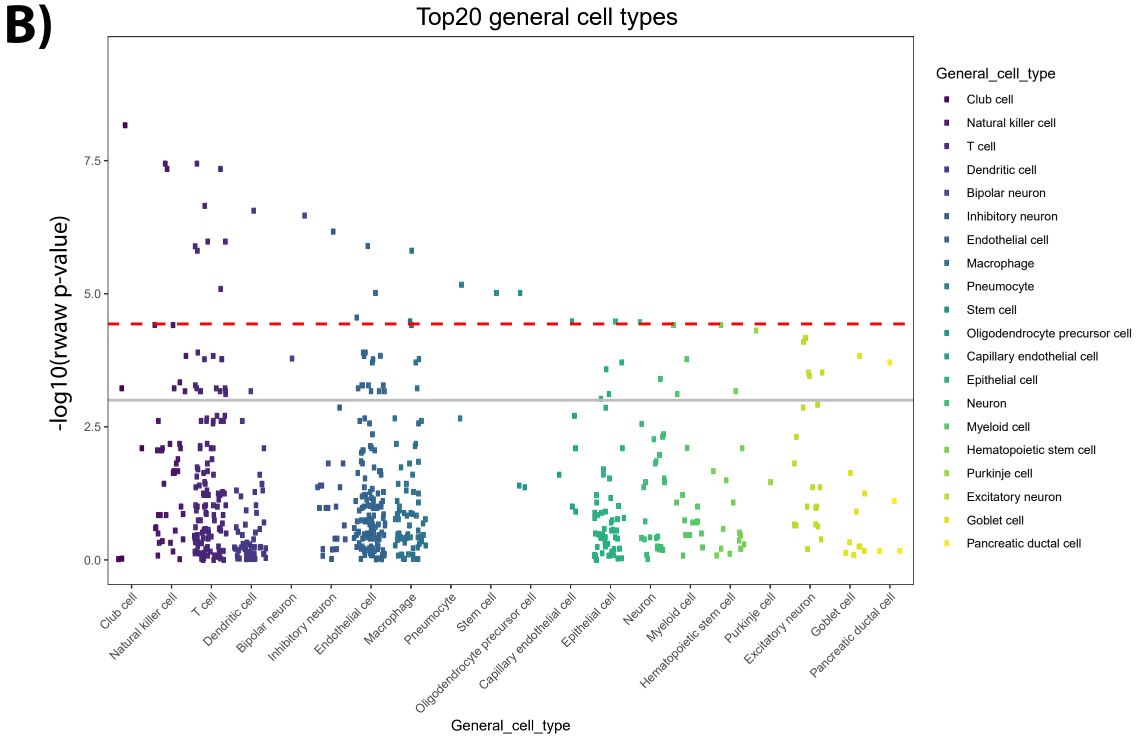

Supplementary Fig. 8. A. WebCSEA top enriched adult organ systems. **B.** WebCSEA top enriched cell-types.

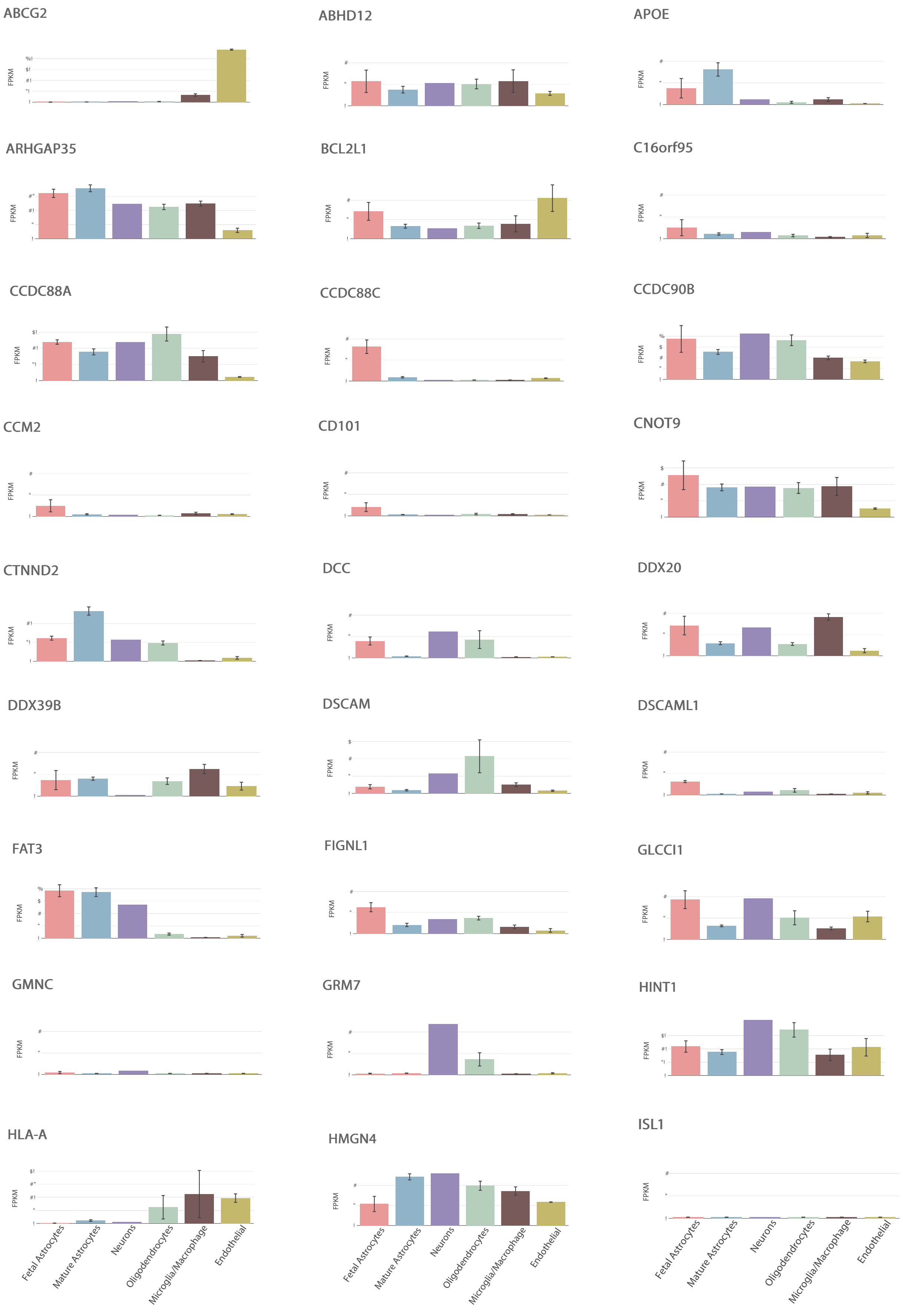
ABCG2
ABHD12
APOE
ARHGAP35
BCL2L1
C16orf95
CCDC88A
CCDC88C
CCDC90B
CCM2
CD101
CNOT9
CTNND2
DCC
DDX20
DDX39B
DSCAM
DSCAML1
FAT3
FIGNL1
GLCCI1
GMNC
GRM7
HINT1
HLA-A
HMGN4
ISL1
FPKM
Fetal Astrocytes
Mature Astrocytes
Neurons
Oligodendrocytes
Microglia/Macrophage
Endothelial

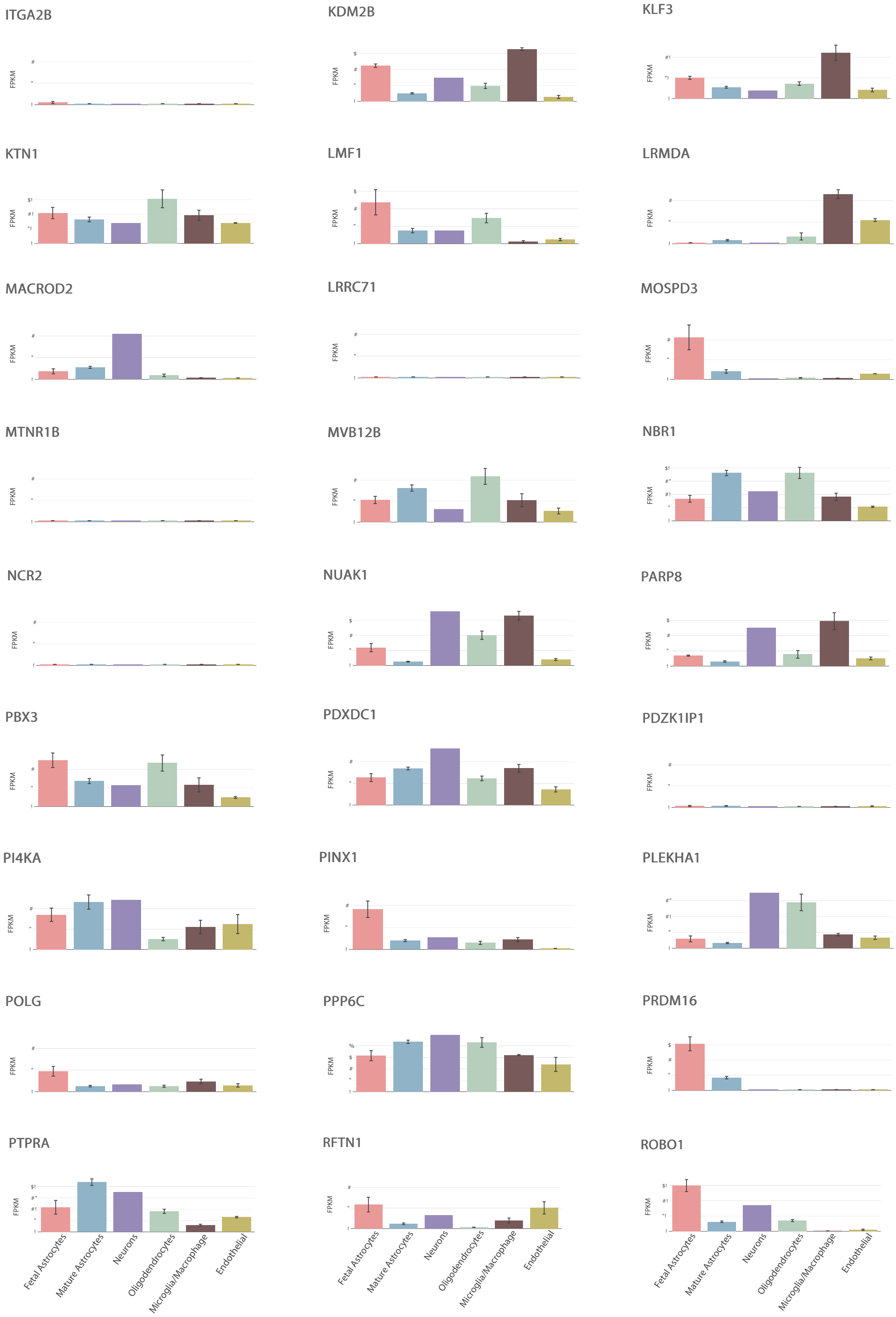
ITGA2B
KDM2B
KLF3
KTN1
LMF1
LRMDA
MACROD2
LRRC71
MOSPD3
MTNR1B
MVB12B
NBR1
NCR2
NUAK1
PARP8
PBX3
PDXDC1
PDZK1IP1
PI4KA
PINX1
PLEKHA1
POLG
PPP6C
PRDM16
PTPRA
RFTN1
ROBO1
FPKM
Fetal Astrocytes
Mature Astrocytes
Neurons
Oligodendrocytes
Microglia/Macrophage
Endothelial

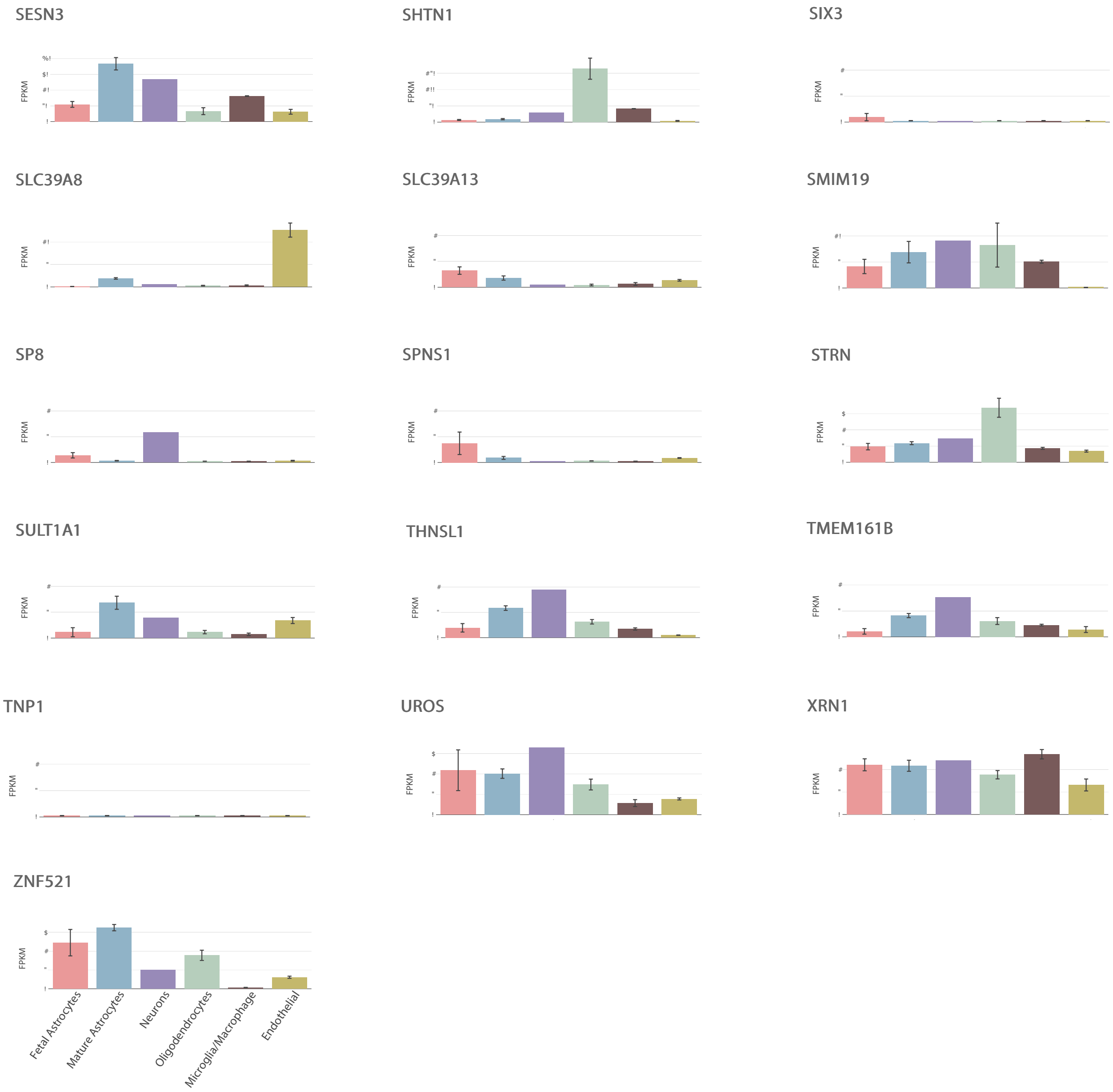

Supplementary Fig. 9. Cell type analysis for the 73 mapped genes. Genes that were not expressed or that weremissing in the data base were not included yielding 70 genes. Panels shows profiles per gene. FPKM=Fragments Per Kilobase Million.

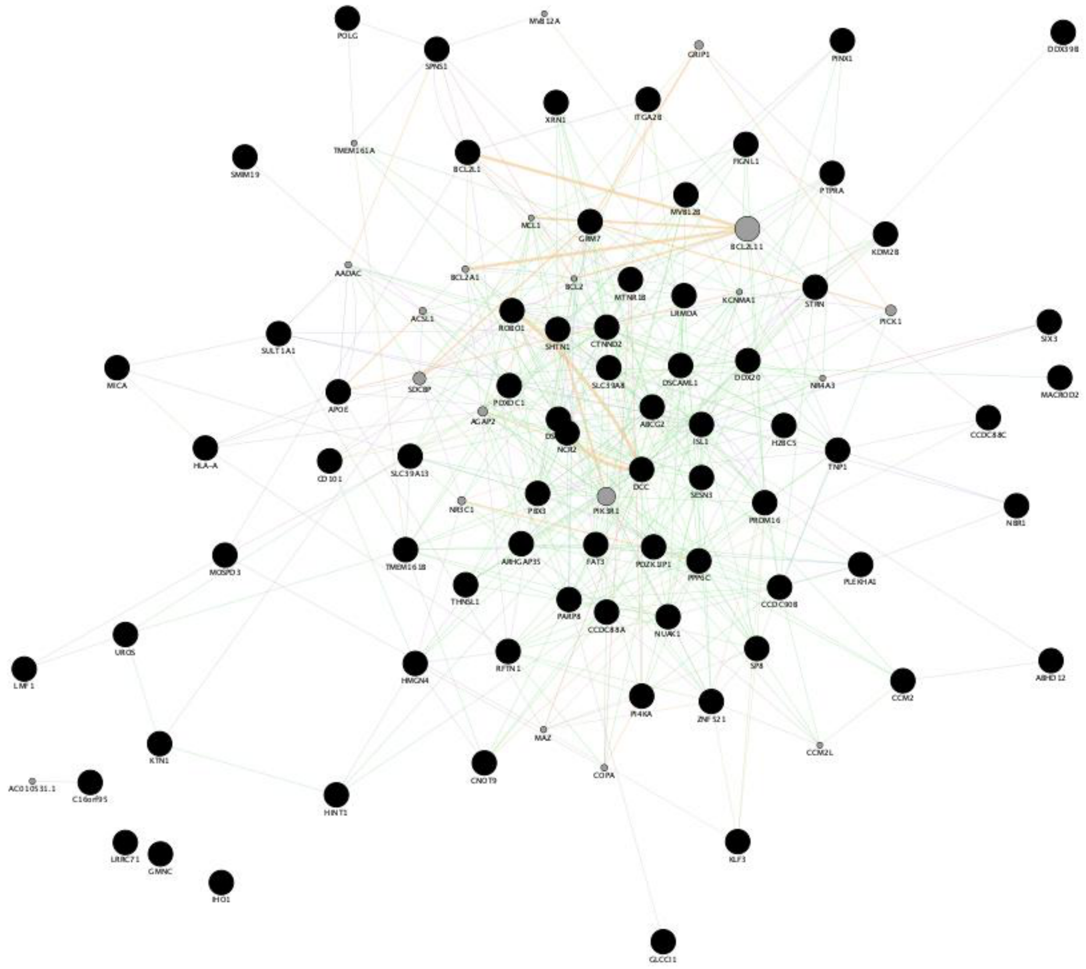

Supplementary Fig. 10. Network interaction graph predominantly illustrating co-expression and shared protein domains for the 74 mapped genes.

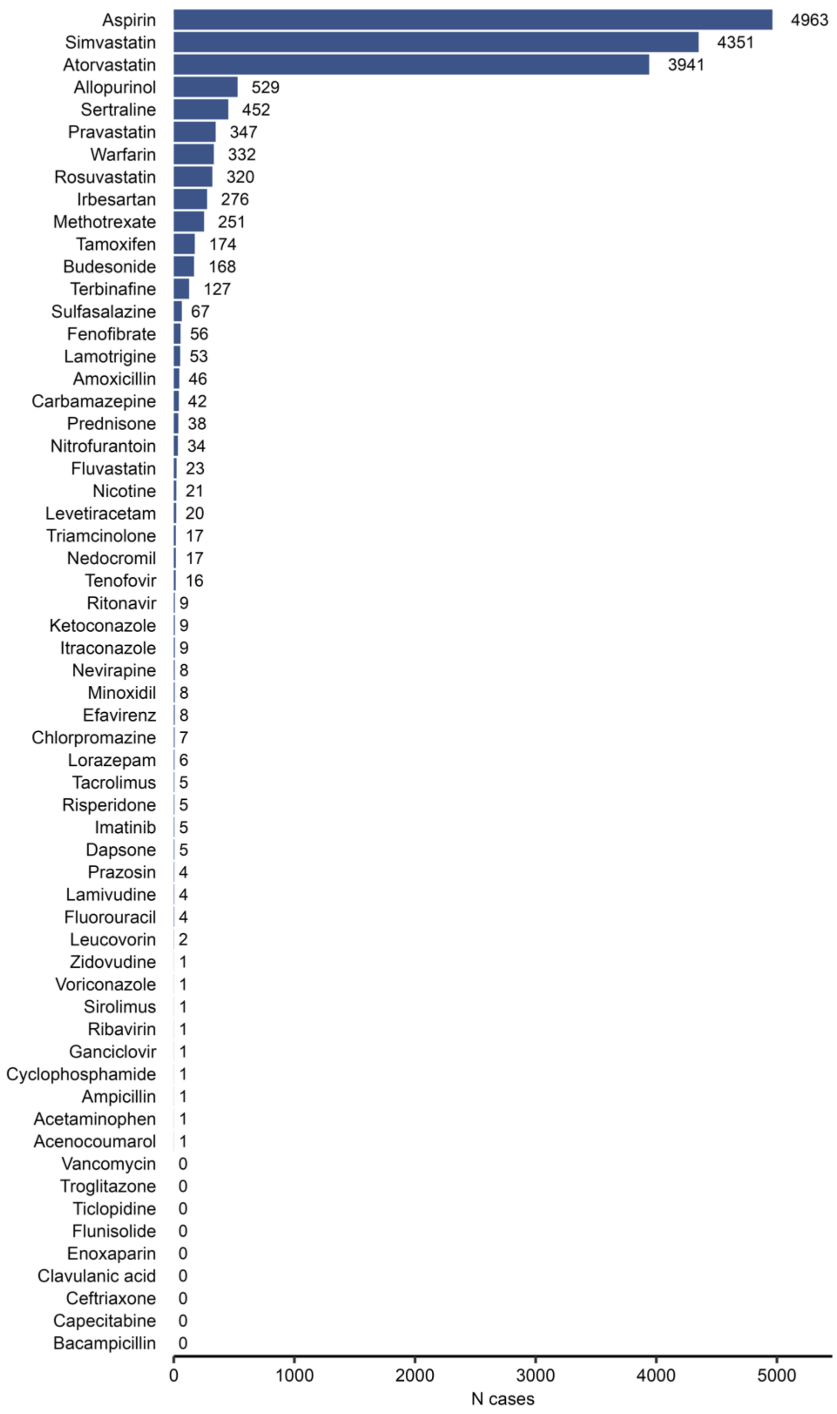

Supplementary Fig. 11. Amount of individuals using any of the 60 drugs both being part of the drug-gene analysis and for which drug use information is available within the UK Biobank.

In our cohort of 34,794 individuals, 4963 are registered as currently or previous users of aspirin, 4351 with simvastatin, 3941 with atorvastatin, 529 using allopurinol, 452 sertraline, 347 using pravastatin, 332 using warfarin, 320 using rosuvastatin, 276 using irbesartan, 251 using methotrexate, 174 using tamoxifen, 168 budesonide, 127 using terbinafine, 67 using sulfasalazine, 56 using fenofibrate, 53 using lamotrigine, 46 using amoxicillin, 42 using carbamazepine, 38 using prednisolone, 34 using nitrofurantoin, 23 using Fluvastatin, 21 using nicotine , 20 using levetiracetam, 17 using triamcinolone, 17 using nedocromil, 16 using tenofovir, 9 using ritonavir, ketoconazole and intraconazole, 8 using nevirapine, minoxidil and efavirenz, 7 using chlorpromazine, 6 using lorazepam, 5 using tacrolimus, risperidone, imatinib and dabsone, 4 using prazosin, lamivudine and fluorouracil, 2 using leucovorin, 1 using zidovudine, virconazole, sirolimus, ribavirin, ganciclovir, cyclophosphamide, apicillin, acetaminophen and acenocoumarol and finally no registred users of vancomycin, troglitazone, ticlopidine, flunisolide, enoxaparin, clavulanic acid, seftriaxone, capecitabine and bacampicillin.

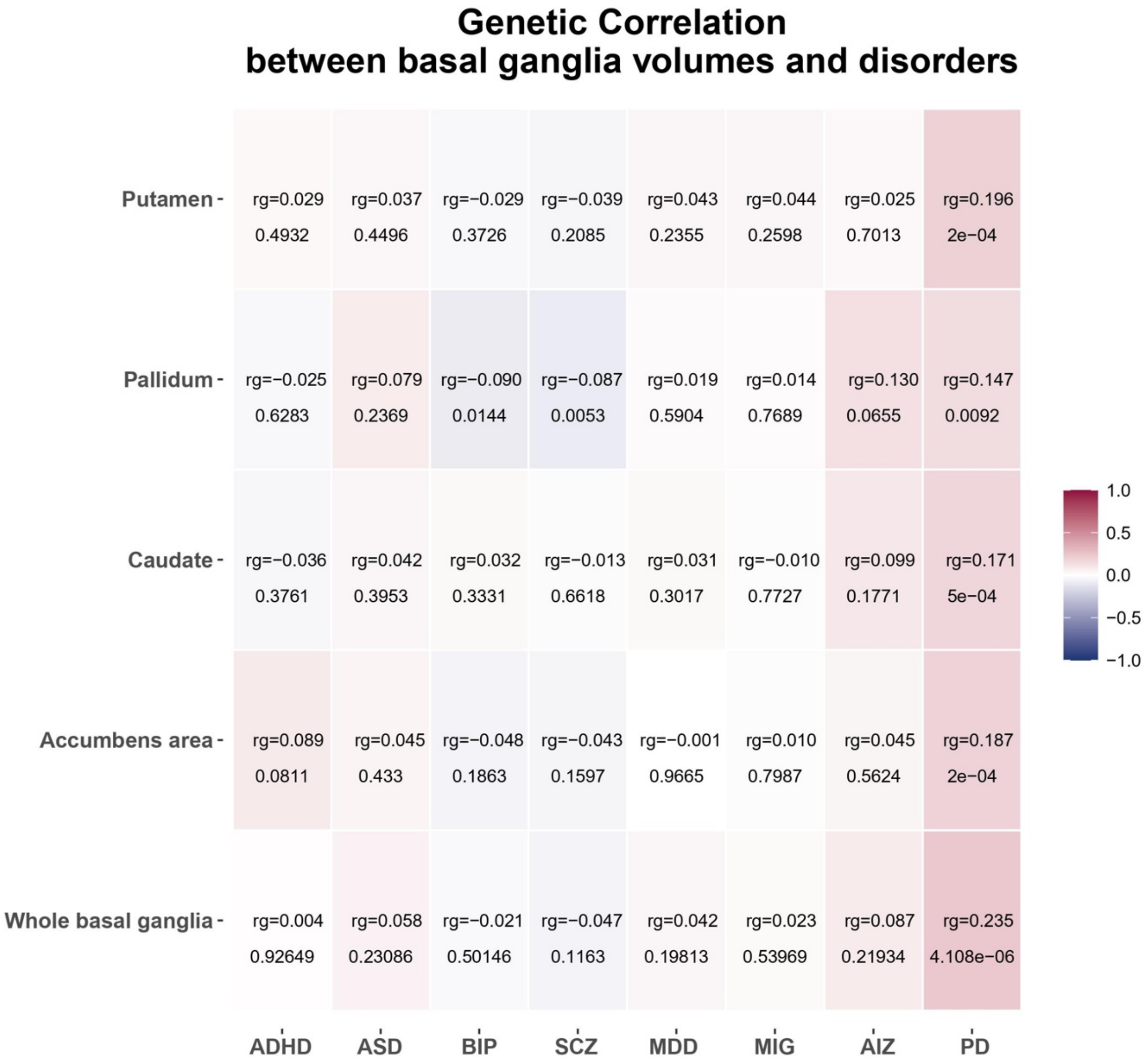

Supplementary Fig. 12. LD-score regression based genetic correlations between basal ganglia volumes and eight brain disorders. The analysis is based on the univariate statistics of the individual regions. Colors reflect correlation strengths. P-values are two-tailed. ADHD; attention-deficit hyperactivity disorder. ASD, autism spectrum disorder; BIP, bipolar disorder; SCZ, schizophrenia; MDD, major depression; MIG: migraine; Alz; Alzheimer's disease; PD, Parkinson's disease.

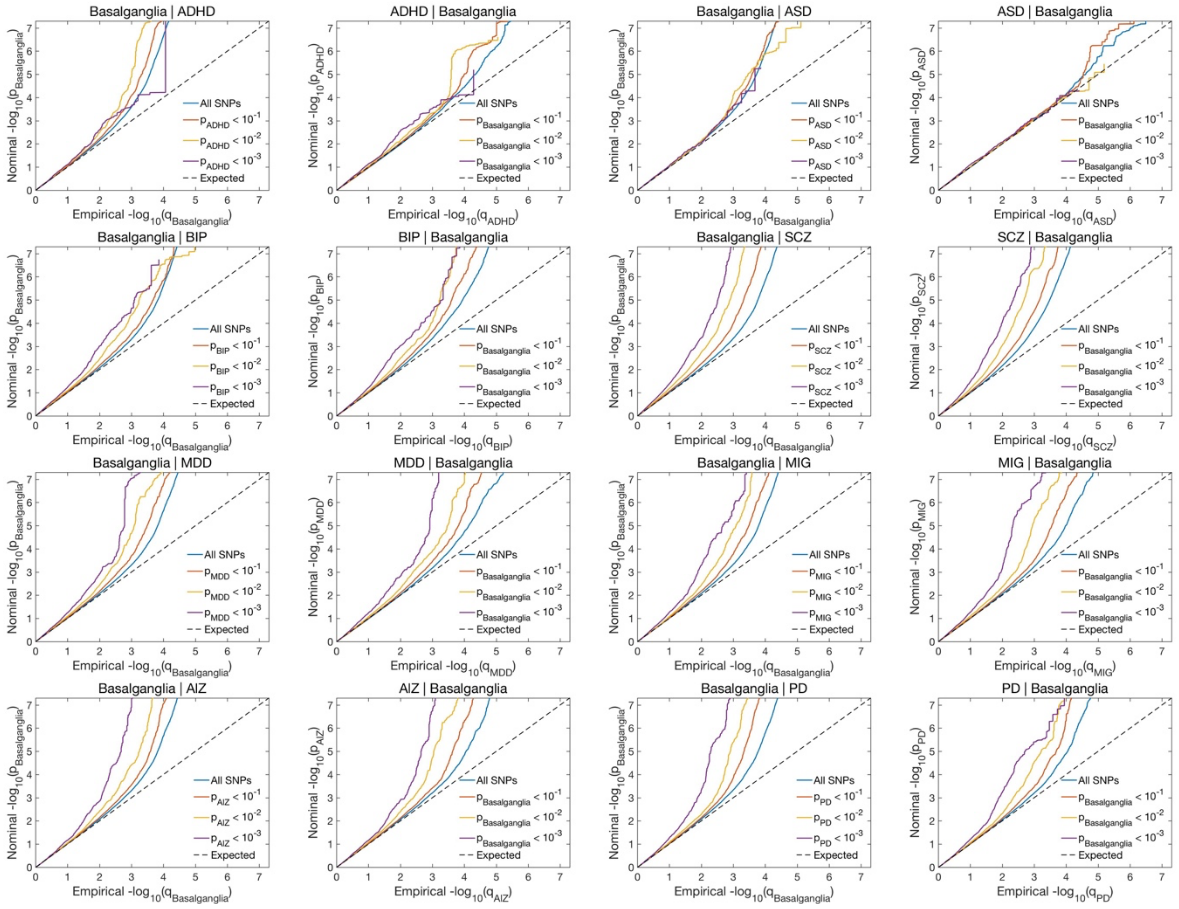

Supplementary Fig. 13. Conditional Q-Q plots for basal ganglia on the brain disorders (left) and vice versa (right). The conditional Q-Q plot shows the relation between expected (x axis) and observed (y axis) significance of variants in the primary phenotype when variants are stratified by their *p*-values in the conditional phenotype. A sequence of four nested strata is presented: all SNPs (blue), $p_{conditional\_phenotype} < 0.1$ (red), $p_{conditional\_phenotype} < 0.01$ (orange) and $p_{conditional\_phenotype} < 0.001$ (purple). Dashed black line demonstrates expected behaviour under no association. Observed significant leftward shift for the group of SNPs with higher significance indicated genetic enrichment and possible shared genetic background between basal ganglia and the brian disorders and vice versa. ADHD; attention-deficit hyperactivity disorder. ASD, autism spectrum disorder; BIP, bipolar disorder; SCZ, schizophrenia; MDD, major depression; MIG: migraine; AD; Alzheimer's disease; PD, Parkinson's disease.

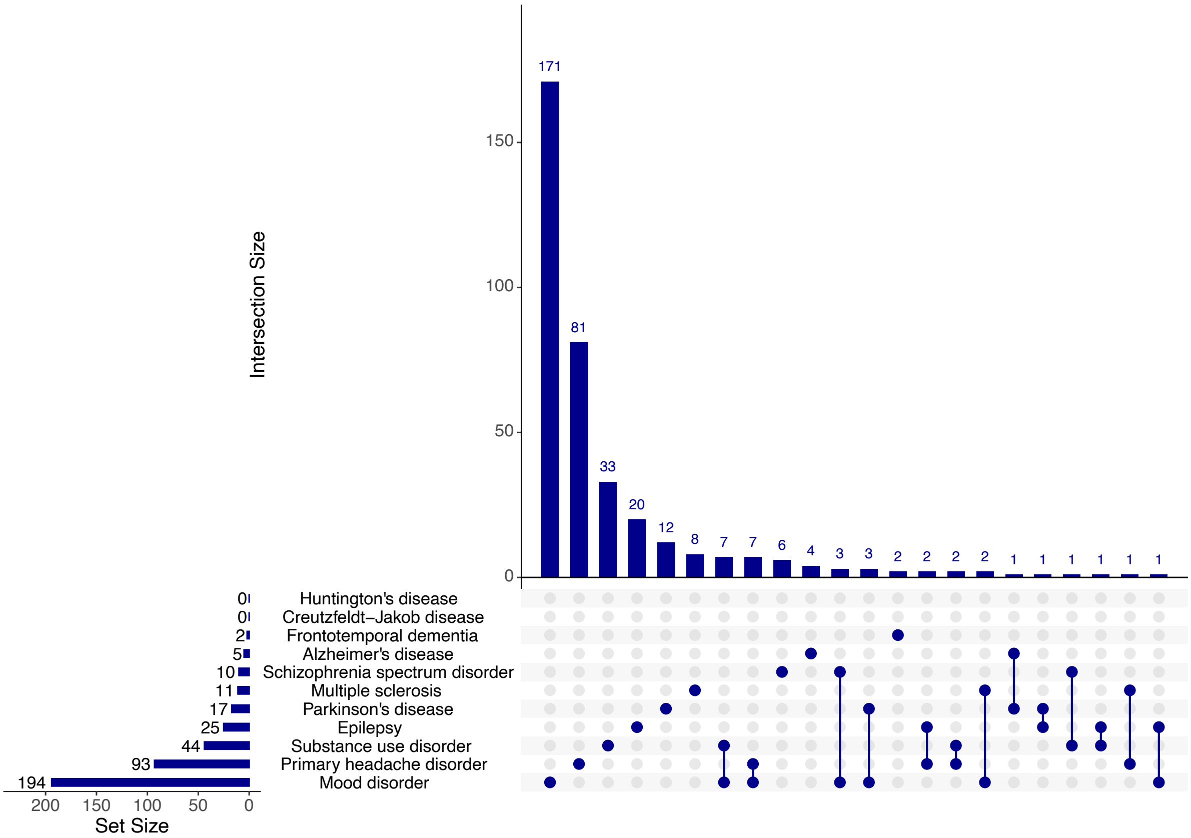

Supplementary Fig. 14. Within our cohort of 34,794 individuals, only a few have registered major neurological and psychiatric conditions. To elaborate, 1477 individuals have recorded mood disorders, with 1423 having experienced major depression and 82 diagnosed with bipolar disorder. Primary headache disorder is noted in 603 individuals, with 535 of them specifically diagnosed with migraine. Schizophrenia spectrum disorder was identified in 50 patients, ADHD in one individual, ASD in 17 individuals, epilepsy in 267, MS in 110, PD in 876, and AD in 52. Diagnoses according to the ICD-10 framework are registered for all individuals previously being, but outpatient treatments lack such documentation.
