# Supplementary material for: The genetic landscape of basal ganglia and implications for common brain disorders
Source: Nat Commun. 2024 Oct 1;15:8476. doi: 10.1038/s41467-024-52583-0 (PMC11445552; doi:10.1038/s41467-024-52583-0)
Supplement: Supplementary file 3 — Description of Additional Supplementary Files [file 41467_2024_52583_MOESM3_ESM.pdf]

Description of Additional Supplementary Files

File Name: Supplementary Data 1
Description: Univariate genome-wide associaton study (GWAS) in 34,794 genotyped white British from the UK Biobank aged 45-82 years identified 11,26,13,23,22 genetic loci associated with Accumbens, Caudate, Pallidum, Putamen and whole basal ganglia regions, respectively (two-sided $P < 5e-8$). The GWAS accounted for age, age squared, sex, scanning site, a proxy of image quality, intracranial volume and the first 20 genetic principal components to control for population stratification. Only p-values below the level of genome-wide significance are reported.

File Name: Supplementary Data 2
Description: The table reports genetic correlations between each combination of volumes after Bonferroni correction.

File Name: Supplementary Data 3
Description: Multivariate genome-wide association study (GWAS) in 34,794 genotyped white British from the UK Biobank aged 45-82 years identified 72 genetic loci associated with basal ganglia (two-sided $P < 5e-8$). The GWAS accounted for age, age squared, sex, scanning site, a proxy of image quality, intracranial volume and the first 20 genetic principal components to control for population stratification. We also display whether the loci were identified in the previous GWASs of the basal ganglia (last column).

File Name: Supplementary Data 4
Description: We functionally annotated all candidate single-nucleotide polymorphisms (SNPs) that were in linkage disequilibrium ($r2 \geq 0.6$) with one of the independent significant SNPs using Functional Mapping and Annotation of GWAS (FUMA). FUMA is based on information from 18 biological repositories and tools and functionally annotates GWAS results. The platform prioritizes the most likely causal SNPs and genes by combining positional, eQTL, and chromatin interaction mapping. FUMA annotates significantly associated SNPs with functional categories, combined CADD scores, RegulomeDB scores, and chromatin states.

File Name: Supplementary Data 5
Description: Genome-wide gene-based association studies (GWGAS; Bonferroni-corrected two-sided $P < 2.622e-6$, i.e 0.05/19073) identified 149 unique genes across the basal ganglia.

File Name: Supplementary Data 6
Description: Genome-wide gene-based associaton studies (GWGAS; Bonferroni-corrected two-sided $P < 2.622e-6$, i.e 0.05/19073) identified 149 unique genes across the hippocampal formation. MAGMA gene-set analysis is performed for curated gene sets and GO terms obtained from MsigDB.

File Name: Supplementary Data 7
Description: We used Open target mapping platform to map the 72 independent significant single-nucleotide polymorphisms (with two-sided $P < 5e-8$) in the GWAS discovery sample to genes. Only p-values below the level of genome-wide significance are reported. This strategy identified 74 unique genes. The pathway analysis was implicated for all mapped genes of basal ganglia volumes.

File Name: Supplementary Data 8
Description: We used Open target mapping platform to map the 72 independent significant single-nucleotide polymorphisms (with two-sided P < 5e-8) in the GWAS discovery sample to genes. Only p-values below the level of genome-wide significance are reported. This strategy identified 74 unique genes. The pathway analysis was implicated for all mapped genes of basal ganglia volumes.

File Name: Supplementary Data 9
Description: We used Open target mapping platform to map the 72 independent significant single-nucleotide polymorphisms (with two-sided P < 5e-8) in the GWAS discovery sample to genes. This strategy identified 74 unique genes. The gene-drug interaction analysis was implicated for all mapped genes of basal ganglia volumes.

File Name: Supplementary Data 10
Description: The table reports correlations between all individual regions and disorders after Bonferroni correction.

File Name: Supplementary Data 11
Description: The most strongly associated lead SNPs in independent genomic loci are shown after merging regions < 250 KB apart into a single locus. Function = functional variant classification based on position in or outside of a gene; CADD = Combined Annotation-Dependent depletion score, which predict how deleterious the SNP effect is on protein structure/function (higher scores indicate more deleterious); RegulomeDB scores predict likelihood of regulatory functionality (lower scores indicate higher likelihood); NA = not annotated; minChrState = minimum chromatin state across 127 tissue types (lower scores indicate more open chromatin). Also shown are p-values and effect sizes (z-scores) from the original summary statistics. In addition, we display results from gene-mapping analyses, including mapping based on 1) SNPs' physical position (postMapFilt), 2) eQTL functionality (eqtlMapFilt) and 3) chromatine interactions (ciMapFilt). Overlap= Overlapped genomic loci associated with basal ganglia and other diseases at conjFDR<0.05.

File Name: Supplementary Data 12
Description: The most strongly associated lead SNPs in independent genomic loci are shown after merging regions < 250 KB apart into a single locus. Function = functional variant classification based on position in or outside of a gene; CADD = Combined Annotation-Dependent depletion score, which predict how deleterious the SNP effect is on protein structure/function (higher scores indicate more deleterious); RegulomeDB scores predict likelihood of regulatory functionality (lower scores indicate higher likelihood); NA = not annotated; minChrState = minimum chromatin state across 127 tissue types (lower scores indicate more open chromatin). Also shown are p-values and effect sizes (z-scores) from the original summary statistics. In addition, we display results from gene-mapping analyses, including mapping based on 1) SNPs' physical position (postMapFilt), 2) eQTL functionality (eqtlMapFilt) and 3) chromatine interactions (ciMapFilt). Overlap= Overlapped genomic loci associated with basal ganglia and other diseases at conjFDR<0.05.

File Name: Supplementary Data 13
Description: The most strongly associated lead SNPs in independent genomic loci are shown after merging regions < 250 KB apart into a single locus. Function = functional variant classification based on position in or outside of a gene; CADD = Combined Annotation-Dependent depletion score, which predict how deleterious the SNP effect is on protein

structure/function (higher scores indicate more deleterious); RegulomeDB scores predict likelihood of regulatory functionality (lower scores indicate higher likelihood); NA = not annotated; minChrState = minimum chromatin state across 127 tissue types (lower scores indicate more open chromatin). Also shown are p-values and effect sizes (z-scores) from the original summary statistics. In addition, we display results from gene-mapping analyses, including mapping based on 1) SNPs' physical position (postMapFilt), 2) eQTL functionality (eqtlMapFilt) and 3) chromatine interactions (ciMapFilt). Overlap= Overlapped genomic loci associated with basal ganglia and other diseases at conjFDR<0.05.

File Name: Supplementary Data 14
Description: The most strongly associated lead SNPs in independent genomic loci are shown after merging regions < 250 KB apart into a single locus. Function = functional variant classification based on position in or outside of a gene; CADD = Combined Annotation-Dependent depletion score, which predict how deleterious the SNP effect is on protein structure/function (higher scores indicate more deleterious); RegulomeDB scores predict likelihood of regulatory functionality (lower scores indicate higher likelihood); NA = not annotated; minChrState = minimum chromatin state across 127 tissue types (lower scores indicate more open chromatin). Also shown are p-values and effect sizes (z-scores) from the original summary statistics. In addition, we display results from gene-mapping analyses, including mapping based on 1) SNPs' physical position (postMapFilt), 2) eQTL functionality (eqtlMapFilt) and 3) chromatine interactions (ciMapFilt). Overlap= Overlapped genomic loci associated with basal ganglia and other diseases at conjFDR<0.05.

File Name: Supplementary Data 15
Description: The most strongly associated lead SNPs in independent genomic loci are shown after merging regions < 250 KB apart into a single locus. Function = functional variant classification based on position in or outside of a gene; CADD = Combined Annotation-Dependent depletion score, which predict how deleterious the SNP effect is on protein structure/function (higher scores indicate more deleterious); RegulomeDB scores predict likelihood of regulatory functionality (lower scores indicate higher likelihood); NA = not annotated; minChrState = minimum chromatin state across 127 tissue types (lower scores indicate more open chromatin). Also shown are p-values and effect sizes (z-scores) from the original summary statistics. In addition, we display results from gene-mapping analyses, including mapping based on 1) SNPs' physical position (postMapFilt), 2) eQTL functionality (eqtlMapFilt) and 3) chromatine interactions (ciMapFilt). Overlap= Overlapped genomic loci associated with basal ganglia and other diseases at conjFDR<0.05.

File Name: Supplementary Data 16
Description: The most strongly associated lead SNPs in independent genomic loci are shown after merging regions < 250 KB apart into a single locus. Function = functional variant classification based on position in or outside of a gene; CADD = Combined Annotation-Dependent depletion score, which predict how deleterious the SNP effect is on protein structure/function (higher scores indicate more deleterious); RegulomeDB scores predict likelihood of regulatory functionality (lower scores indicate higher likelihood); NA = not annotated; minChrState = minimum chromatin state across 127 tissue types (lower scores indicate more open chromatin). Also shown are p-values and effect sizes (z-scores) from the original summary statistics. In addition, we display results from gene-mapping analyses, including mapping based on 1) SNPs' physical position (postMapFilt), 2) eQTL functionality (eqtlMapFilt) and 3) chromatine interactions (ciMapFilt). Overlap= Overlapped genomic loci associated with basal ganglia and other diseases at conjFDR<0.05.

File Name: Supplementary Data 17
Description: The most strongly associated lead SNPs in independent genomic loci are shown after merging regions < 250 KB apart into a single locus. Function = functional variant classification based on position in or outside of a gene; CADD = Combined Annotation-Dependent depletion score, which predict how deleterious the SNP effect is on protein structure/function (higher scores indicate more deleterious); RegulomeDB scores predict likelihood of regulatory functionality (lower scores indicate higher likelihood); NA = not annotated; minChrState = minimum chromatin state across 127 tissue types (lower scores indicate more open chromatin). Also shown are p-values and effect sizes (z-scores) from the original summary statistics. In addition, we display results from gene-mapping analyses, including mapping based on 1) SNPs' physical position (postMapFilt), 2) eQTL functionality (eqtlMapFilt) and 3) chromatine interactions (ciMapFilt). Overlap= Overlapped genomic loci associated with basal ganglia and other diseases at conjFDR<0.05.

File Name: Supplementary Data 18
Description: The most strongly associated lead SNPs in independent genomic loci are shown after merging regions < 250 KB apart into a single locus. Function = functional variant classification based on position in or outside of a gene; CADD = Combined Annotation-Dependent depletion score, which predict how deleterious the SNP effect is on protein structure/function (higher scores indicate more deleterious); RegulomeDB scores predict likelihood of regulatory functionality (lower scores indicate higher likelihood); NA = not annotated; minChrState = minimum chromatin state across 127 tissue types (lower scores indicate more open chromatin). Also shown are p-values and effect sizes (z-scores) from the original summary statistics. In addition, we display results from gene-mapping analyses, including mapping based on 1) SNPs' physical position (postMapFilt), 2) eQTL functionality (eqtlMapFilt) and 3) chromatine interactions (ciMapFilt). Overlap= Overlapped genomic loci associated with basal ganglia and other diseases at conjFDR<0.05.

File Name: Supplementary Data 19
Description: Mendelian randomization (inverse variance weighted, weighted median, weighted mode and MR Egger) performed on 34,794 genotyped white British from the UK Biobank aged 45-82 years identified potential causal relationship between accumbens volume and ALZ risk, and PD risk and caudate and putamen volume (BF adjusted $P < 0.05$).

File Name: Supplementary Data 20
Description: Mendelian randomization (PRESSO) performed on 34,794 genotyped white British from the UK Biobank aged 45-82 years identified potential causal relationship between accumbens volume and ALZ risk, and PD risk and caudate volume. After BF correction.

File Name: Supplementary Data 21
Description: A sensitivity analysis for younger individuals to exclude early structural brain changes in prodromal ALZ or prodromal PD. 65-year age cut-off.
